# HLA class II molecule HLA-DRA identifies immuno-hot tumors and predicts the therapeutic response to anti-PD-1 immunotherapy in NSCLC

**DOI:** 10.1186/s12885-022-09840-6

**Published:** 2022-07-06

**Authors:** Jie Mei, Guanyu Jiang, Yundi Chen, Yongrui Xu, Yuan Wan, Ruo Chen, Feng Liu, Wenjun Mao, Mingfeng Zheng, Junying Xu

**Affiliations:** 1 Department of Oncology, The Affiliated Wuxi People’s Hospital of Nanjing Medical University, No.299, Qingyang Road, Wuxi, 214023 China; 2 Department of Cardiothoracic Surgery, The Affiliated Wuxi People’s Hospital of Nanjing Medical University, No.299, Qingyang Road, Wuxi, 214023 China; 3 The Pq Laboratory of BiomeDx/Rx, Department of Biomedical Engineering, Binghamton University, Binghamton, NY 13902 USA

**Keywords:** NSCLC, Immunotherapy, PD-1, HLA-DRA, Bioinformatics

## Abstract

**Background:**

Immune checkpoint blockade (ICB) only works well for a certain subset of patients with non-small cell lung cancer (NSCLC). Therefore, biomarkers for patient stratification are desired, which can suggest the most beneficial treatment.

**Methods:**

In this study, three datasets (GSE126044, GSE135222, and GSE136961) of immunotherapy from the Gene Expression Omnibus (GEO) database were analyzed, and seven intersected candidates were extracted as potential biomarkers for ICB followed by validation with The Cancer Genome Atlas (TCGA) dataset and the in-house cohort data.

**Results:**

Among these candidates, we found that human leukocyte antigen-DR alpha (HLA-DRA) was downregulated in NSCLC tissues and both tumor and immune cells expressed HLA-DRA. In addition, HLA-DRA was associated with an inflamed tumor microenvironment (TME) and could predict the response to ICB in NSCLC. Moreover, we validated the predictive value of HLA-DRA in immunotherapy using an in-house cohort. Furthermore, HLA-DRA was related to the features of inflamed TME in not only NSCLC but also in most cancer types.

**Conclusion:**

Overall, HLA-DRA could be a promising biomarker for guiding ICB in NSCLC.

**Supplementary Information:**

The online version contains supplementary material available at 10.1186/s12885-022-09840-6.

## Background

Lung cancer is the most common malignancy, and non-small cell lung cancer (NSCLC) accounts for approximately 85% of lung cancers [1]. The prognosis of patients with lung cancer is significantly poor [2]. The treatment landscape for advanced NSCLC has been changing rapidly. Recent advancements in the molecular profiling of NSCLC enable targeted molecular therapy with EGFR tyrosine kinase inhibitors (TKI) and immune checkpoint inhibitors [3, 4]. Accordingly, patients with EGFR-mutated NSCLC have achieved better progression-free survival (PFS) and overall survival (OS). On the other hand, patients harboring wild-type EGFR and overexpressed PD-L1 may benefit from immune checkpoint blockade (ICB) therapy [5].

As one of the significant features of malignant tumors, immune escape plays an important role in the oncogenesis, progression, and therapeutic resistance of malignant tumors [6]. In the past decade, immunotherapy targeting immune checkpoints has opened up a new path for treating malignant tumors [7]. Thus far, immunotherapy drugs represented by PD-1/PD-L1 inhibitors have been widely used for treating a variety of malignant tumors, such as NSCLC, triple-negative breast cancer, melanoma, and Hodgkin lymphoma [8–11]. Among these cancer types, immunotherapy is the most widely used in NSCLC. However, not all patients could benefit from immunotherapy. Therefore, appropriate biomarkers to identify the dominant population are very critical in clinical application.

Currently, the expression of PD-L1 in tumor cells is considered the most reasonable biomarker for selecting the dominant population in anti-PD-1 immunotherapy. The results of multiple clinical trials in advanced NSCLC support the effectiveness of positive expression of PD-L1 in tumor cells in predicting the efficacy of immunotherapy [5, 12, 13]. Nevertheless, CheckMate017 and OAK studies reported that patients with negative PD-L1 expression can also benefit from immunotherapy [14, 15]. In summary, it can be found that the PD-L1 expression level of tumor cells is unreliable as a biomarker for predicting the efficacy of immunotherapy [16, 17]. Our previous studies have been devoted to the study of immunotherapy biomarkers, and proposed that deglycosylated PD-L1 and IFITM3 could be potential biomarkers for immunotherapy [18, 19].

This study aimed to identify novel therapeutic biomarkers for immunotherapy in NSCLC. First, we collected three public datasets of immunotherapy from the Gene Expression Omnibus (GEO) database and screened potential biomarkers for immunotherapy. Given the expression patterns and prognostic values of intersected candidates, human leukocyte antigen-DR alpha (HLA-DRA) was selected for further analysis and validation with the Cancer Genome Atlas (TCGA) dataset and the in-house cohort. Overall, our findings demonstrated that HLA-DRA could be a novel biomarker in NSCLC, which may make up for the deficiency of PD-L1 in the selection of the dominant population of immunotherapy.

## Methods

### Acquisition of public data

The preprocessed RNA-sequencing (RNA-seq) data and immunotherapeutic responses of GSE126044 [20], GSE135222 [21], and GSE136961 [22] were downloaded from the GEO database (https://www.ncbi.nlm.nih.gov/geo/). The pan-cancer (TOIL RSEM tpm) and NSCLC (IlluminaHiSeq) gene expression profiles as well as clinical annotations of the TCGA dataset were obtained from the UCSC Xena (https://xenabrowser.net/datapages/). The abbreviations for various cancer types are exhibited in **Supplementary Table S****1**.

### DGEs screening and enrichment analysis

To identify differentially expressed genes (DEGs) between responders and non-responders, student t test was used and genes with *P* value ≤0.05 were deemed to be candidates. Next, common candidates between GSE126044, GSE135222, and GSE136961 datasets were extracted using Venn analysis. To analyze the biological processes (BP) of intersected genes in these three GEO datasets, the DAVID tool was applied. DAVID (https://david.ncifcrf.gov/) is a widely used gene functional annotation tool [23]. The human genome (homo sapiens) was selected as the background variable. BP terms were deemed to be statistically significant when the *P* value was ≤0.05 and the top 10 terms were retained.

### ScRNA-seq analysis

TISCH (http://tisch.comp-genomics.org/gallery/) is a single-cell RNA-sequencing (scRNA-seq) database focusing on the tumor microenvironment (TME) and provides detailed cell-type annotation at the single-cell level [24]. In this research, we used TISCH to analyze the cell subpopulation patterns of seven intersected genes from three GEO datasets in NSCLC. Default options were used for all parameters.

### Immunological correlation of HLA-DRA in NSCLC

The immunological characteristics of TME in NSCLC contained immunomodulators, tumor purity, infiltration levels of tumor-infiltrating immune cells (TIICs), and the expression of inhibitory immune checkpoints. Firstly, we studied the expression of 150 immunomodulators, including MHC, receptors, chemokines, immunoinhibitors, and immunostimulators. In addition, tumor purity was obtained from previous research and its correlation with HLA-DRA was assessed [25]. Moreover, the correlations between HLA-DRA and immune checkpoints levels were evaluated. To avoid the calculation error caused by various algorithms, we used two independent algorithms, including TIMER [26] and EPIC [27] to estimate TIICs abundance. To investigate the associations between HLA-DRA and anti-tumor immunity in NSCLC, we divided the samples into the high and low HLA-DRA groups at its median expression, and then analyzed the differences in immunological characteristics of TME in these aspects.

### Correlation between HLA-DRA and immunotherapeutic response

According to a previous report, immunophenoscore (IPS) was calculated to predict therapeutic response to immunotherapy [28]. The IPS values of NSCLC patients were obtained from the Cancer Immunome Atlas (TCIA) website (http://tcia.at/home/). In addition, we also computed the T cell inflamed score based on the expression and weighting coefficient of 18 genes [29]. Referring to previous research [30], we obtained several gene-sets which were correlated with the immunotherapeutic response as well as specific gene markers of biological processes related to anti-tumor immunity (**Supplementary Table S****2**). The enrichment scores of these gene signatures were calculated utilizing the R package “GSVA” [31]. To evaluate the role of HLA-DRA in predicting immunotherapeutic responses, the associations between HLA-DRA expression and these aspects were evaluated.

### Pan-cancer analysis of immunological correlation of HLA-DRA

To evaluate the pan-cancer immunological correlation of HLA-DRA, we collected pan-cancer expression of 150 immunomodulators, including MHC, receptors, chemokines, immunoinhibitors, and immunostimulators. Then, the correlations between HLA-DRA and tumor purity as well as TIICs abundance were also assessed.

### Clinical samples

The NSCLC tissue microarray (TMA, Cat. HLugA150CS03) and the pan-cancer TMA (Cat. HOrgC120PG04) were purchased from Outdo BioTech (Shanghai, China). A total of 75 NSCLC and para-tumor samples were contained in this research. The HOrgC120PG04 microarray contained 11 kinds of cancers with 2–6 tumor samples and para-tumor or normal samples per type. Ethical approval for the use of TMAs was granted by the Clinical Research Ethics Committee in Outdo Biotech (Shanghai, China). In addition, 16 cancer patients who received anti-PD-1 immunotherapy monotherapy or a combination of chemotherapy were recruited by The Affiliated Wuxi People’s Hospital of Nanjing Medical University. The therapeutic response was evaluated according to the RECIST 1.1 criterion. Among them, 8 patients reached partial response (PR), and 8 patients were stable disease (SD). Ethical approval for the collection of tissue sections was granted by the Clinical Research Ethics Committee, The Affiliated Wuxi People’s Hospital of Nanjing Medical University.

### Immunohistochemistry (IHC) staining and semi-quantitative scoring

IHC staining was conducted on the above sections according to the standardized procedures. Sections were retrieved by EDTA. The primary antibodies used were as follows: anti-HLA-DRA (1:2500 dilution, Cat. 17,221–1-AP, ProteinTech) and anti-PD-L1 (Ready-to-use, Cat. GT2280, GeneTech). Staining was visualized with DAB and hematoxylin counterstain, and stained sections were captured using Aperio Digital Pathology Slide Scanners. For semi-quantitative analysis, the stained sections were independently evaluated by two pathologists according to the evaluation standard on a 12-point scale by calculating the immunoreactivity score (IRS) [32].

### Statistical analysis

All statistical analyses were conducted using SPSS 26.0 software or R language. All data are presented as means ± SDs. The difference between the two groups was analyzed by parametric Student’s t-test or non-parametric Mann Whitney test. Survival analysis was performed by log-rank test. Correlation analysis between two variables was analyzed by Pearson test. All statistical tests were two-sided, and *P* value < 0.05 was considered statistically significant and labeled with *P < 0.05; **P < 0.01; ***P < 0.001.

## Results

### Identification of potential biomarkers for immunotherapy in NSCLC

**Fig. 1:**
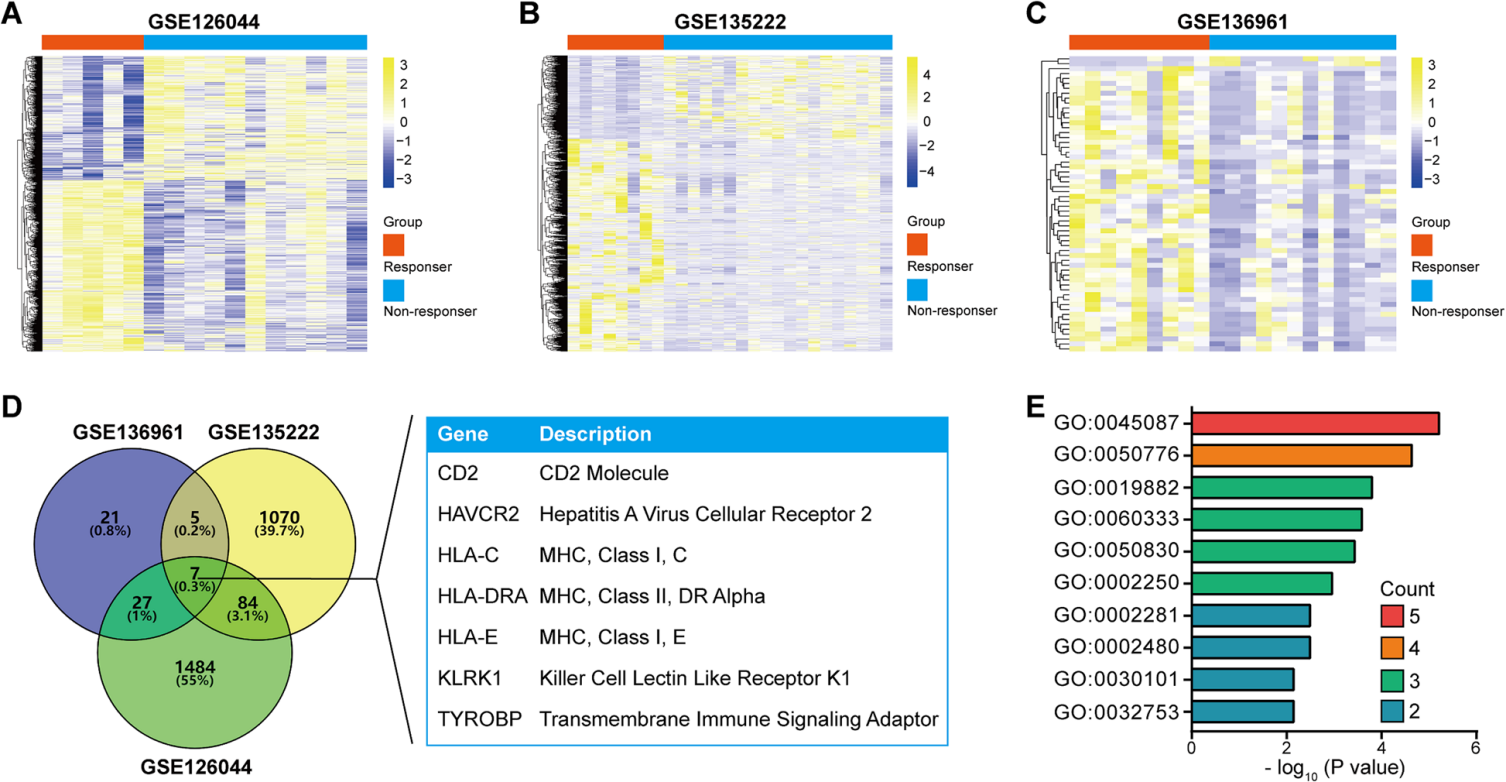
Screening potential biomarkers for immunotherapy in NSCLC. **A** Heatmap of DEGs between samples from responders and non-responders in the GSE126044 dataset. **B** Heatmap of DEGs between samples from responders and non-responders in the GSE13522 dataset. **C** Heatmap of DEGs between samples from responders and non-responders in the GSE136961 dataset. **D** The intersection of DEGs in GSE126044, GSE13522, and GSE136961 datasets. **E** BP analysis of intersected genes in these three GEO datasets. “Count” represents the number of 7 candidates

To discover novel biomarkers to identify the responsive population to the particular immunotherapy, we downloaded GSE126044, GSE135222, and GSE136961 datasets from the GEO database. DEGs were extracted with *P* value ≤0.05. A total of 1605 DEGs in the GSE126044 dataset (Fig. 1A), 1166 DEGs in the GSE135222 dataset (Fig. 1B), and 60 DEGs in the GSE136961 dataset (Fig. 1C) were identified. Subsequently, common candidates in these three datasets were extracted using Venn analysis, and a total of seven genes (CD2, HAVCR2, HLA-C, HLA-DRA, HLA-E, KLRK1, and TYROBP) were selected (Fig. 1D). Next, we analyzed the potential functions of intersected genes using the DAVID tool. The results showed that these genes mainly participated in immune-related processes, such as the innate immune response, regulation of immune response, antigen processing and presentation, etc. (Fig. 1E**, Supplementary Table S****3**). Overall, these findings revealed seven potential biomarkers for immunotherapy in NSCLC.

### Expression, atlas of cell subtypes, and prognostic values of potential biomarkers in NSCLC

**Fig. 2:**
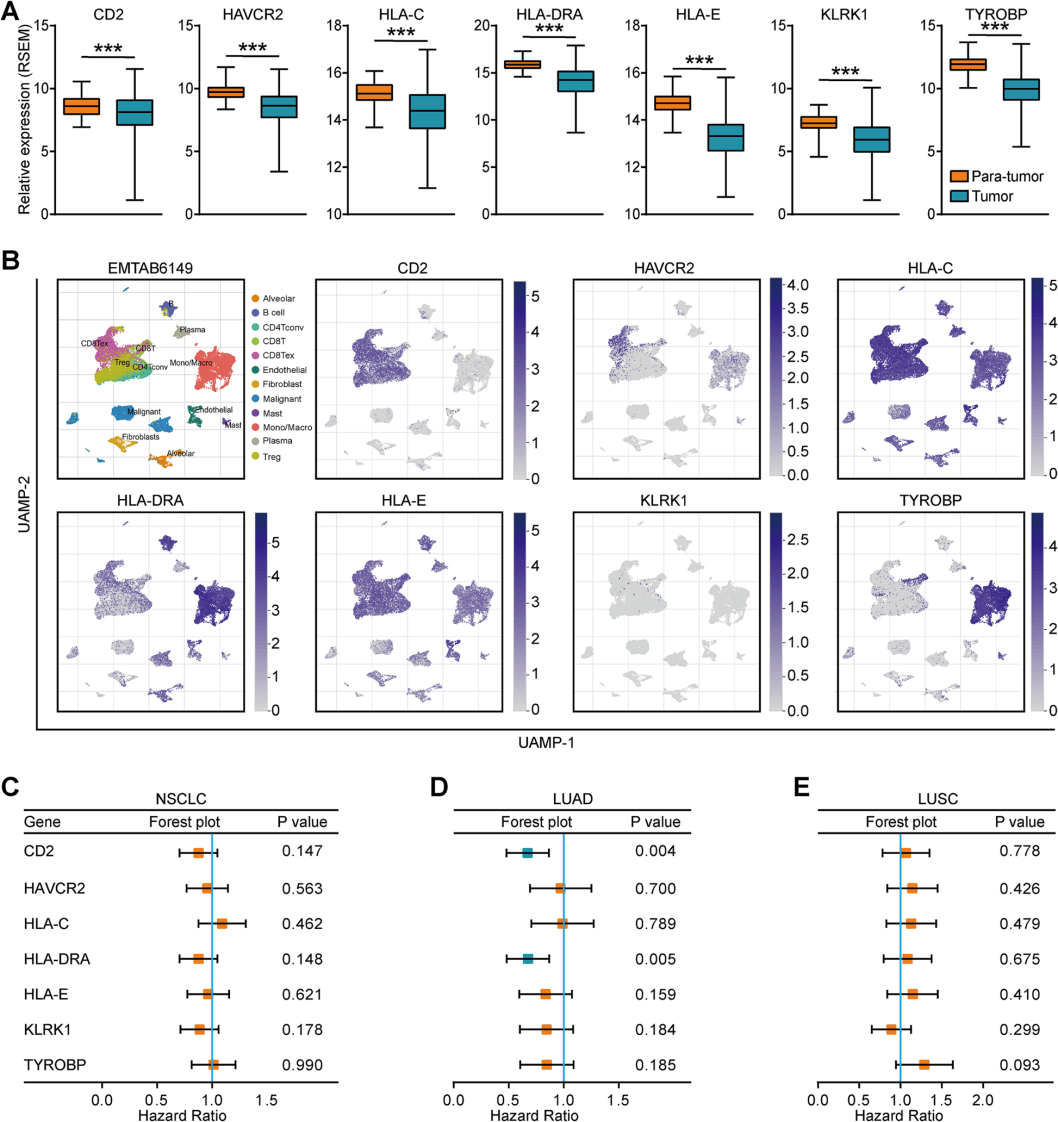
Expression patterns and prognostic values of potential biomarkers. **A** Expression levels of seven candidates in tumor and para-tumor tissues in NSCLC. **B** Cell subsets of seven candidates’ expressions in NSCLC. The data was obtained from the TISCH database. **C** Prognostic values of seven candidates in NSCLC. **D** Prognostic values of seven candidates in LUAD. **E** Prognostic values of seven candidates in LUSC

We analyzed the expression of potential biomarkers in NSCLC. In the TCGA dataset, the expression levels of CD2, HAVCR2, HLA-C, HLA-DRA, HLA-E, KLRK1, and TYROBP were all significantly downregulated in tumor tissues compared with para-tumor tissues (Fig. 2A). We also investigated the atlas of cell subtypes of these candidates. The results showed that CD2 was mainly expressed in CD4+ and CD8+ T cells, HAVCR2 was mainly expressed in exhausted T cells, three HLA molecules were expressed in almost all cell types, TYROBP was mainly expressed in monocytes and macrophages, and KLRK1 was lowly expressed in all cell types (Fig. 2B). As to their prognostic values, seven genes could not predict the clinical outcome in NSCLC and lung squamous cell carcinoma (LUSC), while CD2 and HLA-DRA were associated with the improved prognosis in lung adenocarcinoma (LUAD) (Figs. 2C-E). Moreover, the expression levels of these genes were significantly correlated with each other in GSE126044, GSE135222, and GSE136961 datasets (**Supplementary Figs. S****1****A-C**). In view of the above findings, we selected HLA-DRA for further investigation and validation.

### HLA-DRA is associated with an inflamed TME in NSCLC

**Fig. 3:**
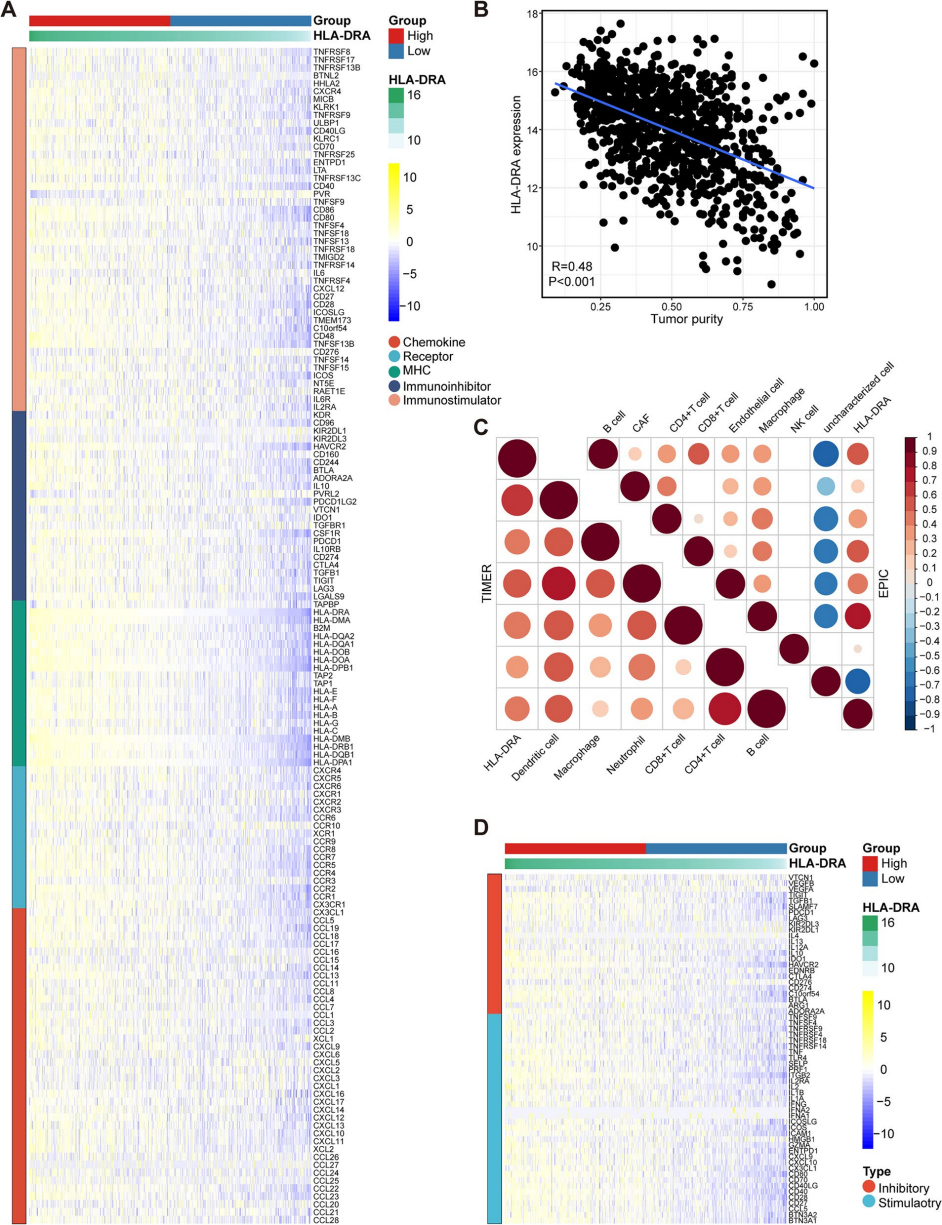
HLA-DRA predicts an inflamed TME in NSCLC. **A** Expression levels of immunomodulators (MHC, receptors, chemokines, immunoinhibitors, and immunostimulators) in the high- and low-HLA-DRA groups in NSCLC. **B** Correlation between HLA-DRA expression and tumor purity in NSCLC. **C** Correlations between HLA-DRA expression and the levels of TIICs calculated using two algorithms. The color and the values indicate the Pearson correlation coefficient. **D** Expression levels of inhibitory and stimulatory immune checkpoints in the high- and low-HLA-DRA groups in NSCLC

**Fig. 4:**
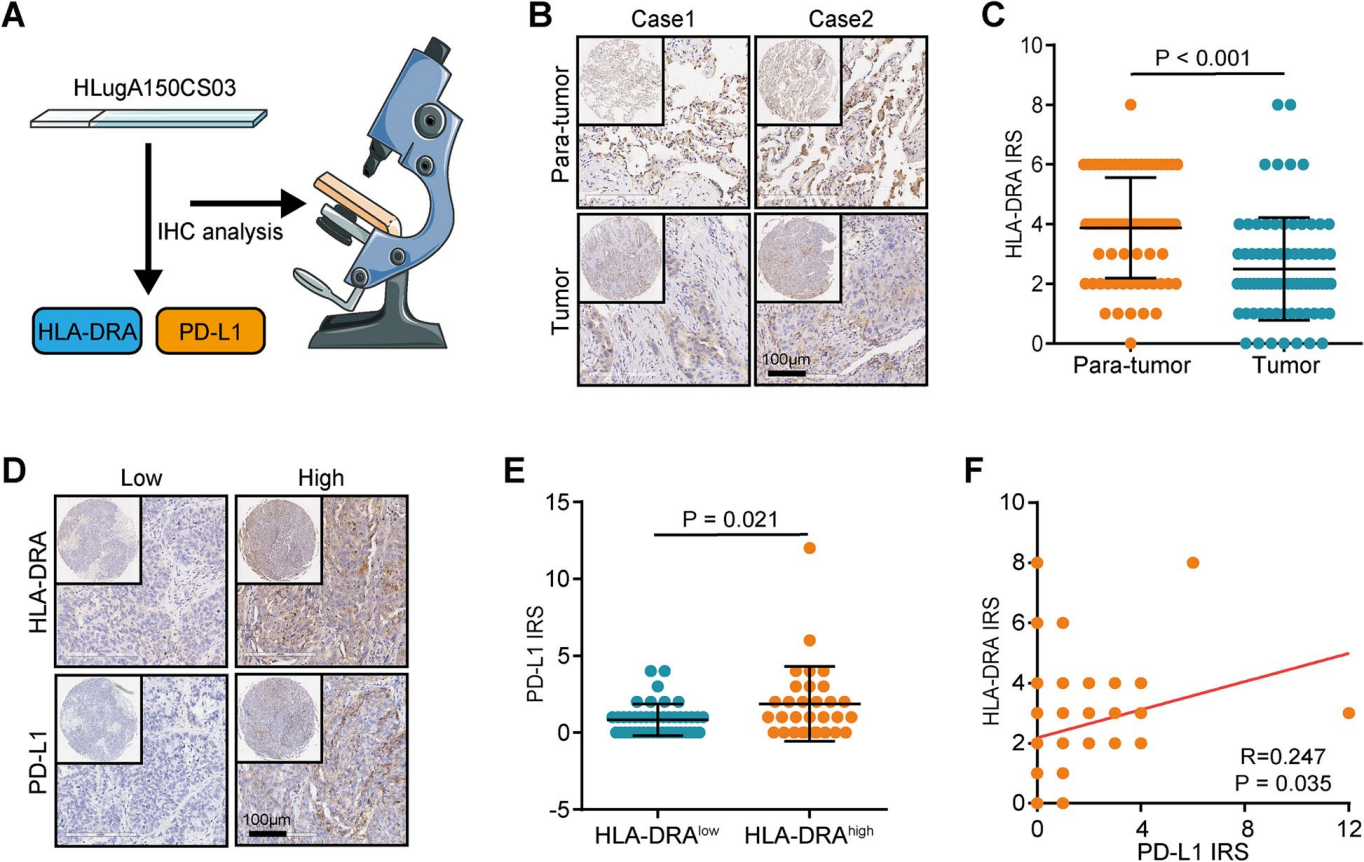
Validation of HLA-DRA expression and its correlation with PD-L1. **A** Schematic protocol of validation on the NSCLC TMA cohort. **B** Representative images revealing HLA-DRA expression in para-tumor and tumor tissues in NSCLC. Magnification, 200×. **C** Expression levels of HLA-DRA in tumor and para-tumor tissues. **D** Representative images revealing PD-L1 expression in the high and low HLA-DRA groups. Magnification, 200×. **E** Differences in PD-L1 expression between the high and low HLA-DRA groups. **F** Correlation between HLA-DRA and PD-L1 expression in the NSCLC TMA cohort

Given the correlation between HLA-DRA and immunotherapeutic response, we explored the immunological role of HLA-DRA in NSCLC using the TCGA cohort. Many chemokines, paired receptors, MHC molecules, immunoinhibitors, and immunostimulators were notably upregulated in the high-HLA-DRA group (Fig. 3A). Considering that these chemokines and receptors recruit effector TIICs, we speculated that HLA-DRA was correlated with increased TIICs in TME. Interestingly, HLA-DRA was negatively correlated with tumor purity (Fig. 3B). Furthermore, HLA-DRA was positively correlated with TIIC abundance estimated by TIMER and EPIC algorithms (Fig. 3C). Moreover, HLA-DRA was positively correlated with expression levels of immune checkpoints in NSCLC (Fig. 3D). To validate the above findings, an NSCLC TMA cohort was used (Fig. 4A). The results showed that the expression of HLA-DRA protein was significantly downregulated in tumor tissues (Figs. 4B-C). Furthermore, the current NSLCL cohort was classified into low- and high-HLA-DRA expression groups based on its median level (IRS ≤ 2 vs. IRS > 3). We found that PD-L1 expression was higher in the high-HLA-DRA group (Figs. 4D-E). Furthermore, HLA-DRA was positively correlated with PD-L1 expression in the NSCLC cohort (Fig. 4F). Overall, HLA-DRA is highly correlated with the inflamed TME and identified immuno-hot tumors in NSCLC.

### HLA-DRA predicts the response to immunotherapy in NSCLC

**Fig. 5:**
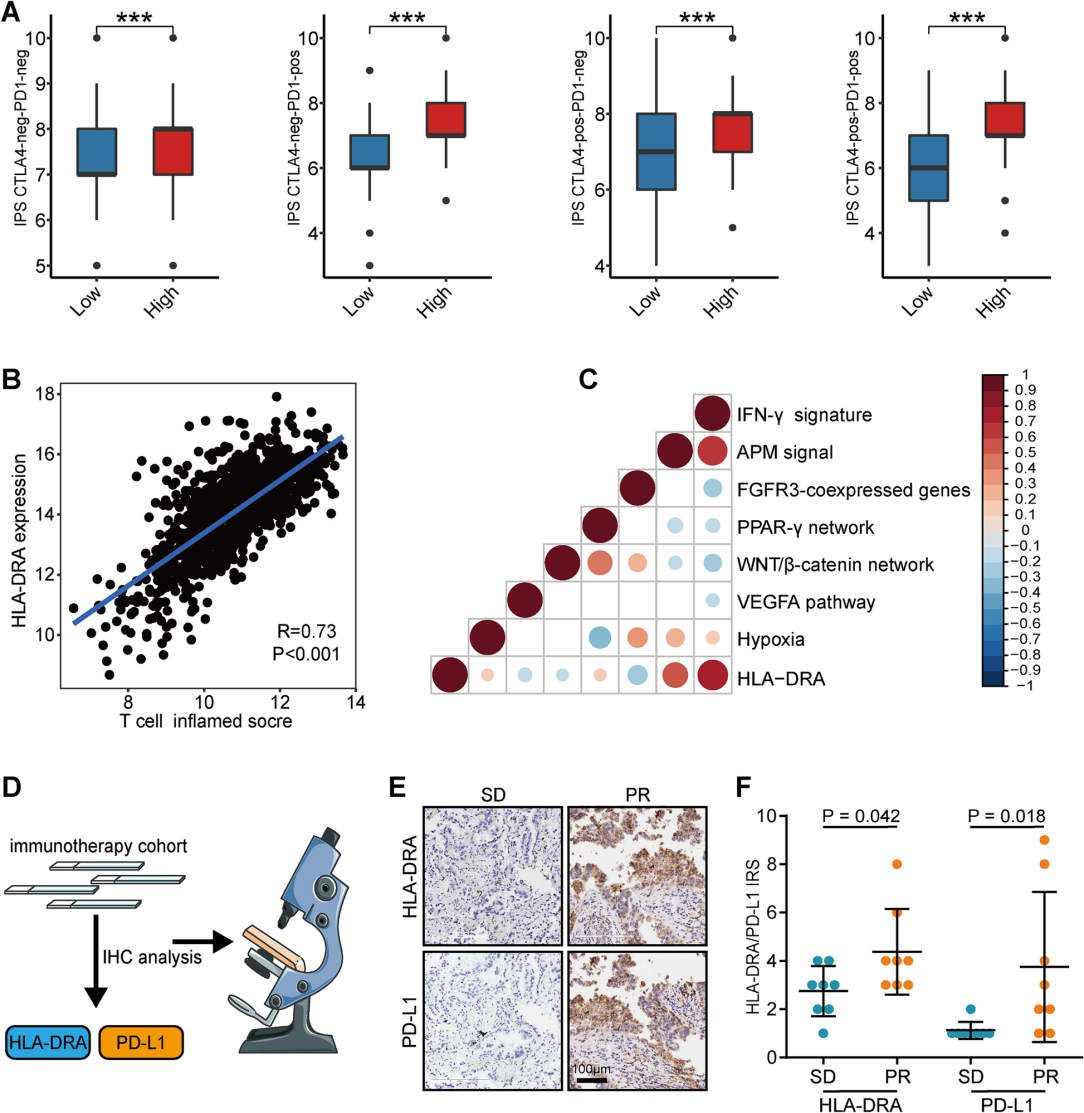
HLA-DRA predicts immunotherapeutic response of immunotherapy. **A** Differences in levels of IPS in the high and low HLA-DRA groups in NSCLC. **B** Correlation between HLA-DRA and T cell inflamed score in NSCLC. **C** Correlation between HLA-DRA and immune-related pathways in NSCLC. **D** Schematic protocol of validation using the immunotherapy cohort. **E** Representative images revealing HLA-DRA and PD-L1 expression in NSCLC tissues with different immunotherapeutic responses. Magnification, 200×. **F** Expression levels of HLA-DRA and PD-L1 in NSCLC patients with immunotherapeutic responses

We further validated whether NSCLC patients with high HLA-DRA expression exhibited high responses to immunotherapy. IPS was a surrogate of the response to immunotherapy, and we found that patients with high HLA-DRA expression had notably high IPS (Fig. 5A). T cell-inflamed score was developed using IFN-γ-related mRNA profiles to predict response to anti-PD-1 therapy. The results showed that HLA-DRA was positively correlated with T cell-inflamed scores in NSCLC (Fig. 5B). Moreover, the immunotherapy-related enrichment scores, such as IFN-γ signature, APM signal, and hypoxia, were positively correlated with HLA-DRA expression, while FGFR3-coexpressed genes, PPAR-γ network, WNT/β-catenin network, and VEGFA pathway were negatively correlated HLA-DRA expression (Fig. 5C). To further validate the association between HLA-DRA expression and immunotherapeutic response, a small-scale immunotherapy cohort was used (Fig. 5D). The results showed that HLA-DRA and PD-L1 were significantly upregulated in the patients with the response of PR than SD (Figs. 5E-F). Collectively, public and in-house cohorts support that HLA-DRA could be a novel biomarker for predicting the response to anti-PD-1 immunotherapy in NSCLC.

### Extension of the immunological role of HLA-DRA in pan-cancer

**Fig. 6:**
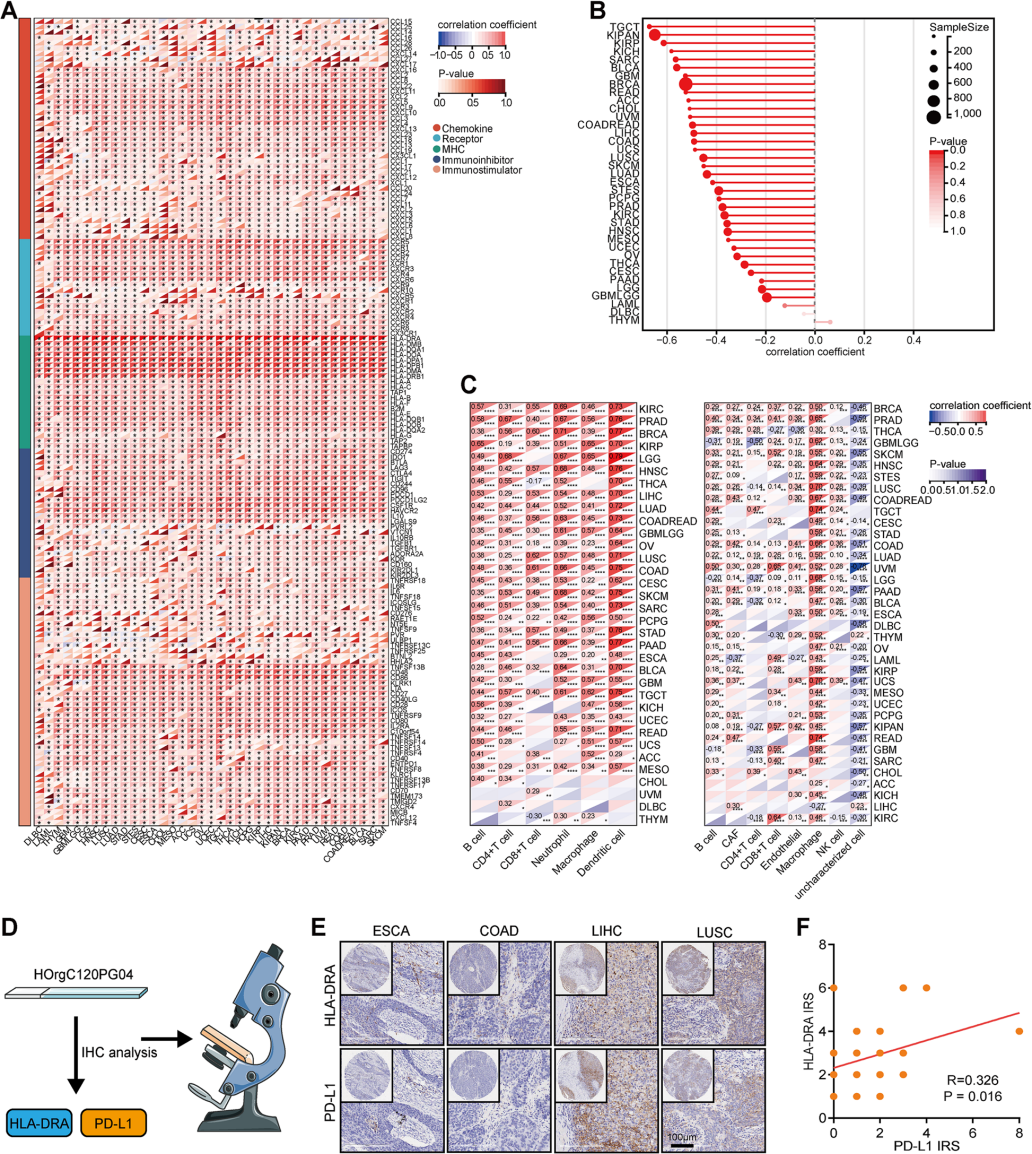
Pan-cancer analysis of the immunocorrelation of HLA-DRA. **A** Correlations between HLA-DRA and 150 immunomodulators (MHC, receptors, chemokines, immunoinhibitors, and immunostimulators) in pan-cancer. The color indicates the correlation coefficient. The asterisks indicate significant differences assessed by Pearson analysis. **B** Correlations between HLA-DRA and tumor purity in pan-cancer. **C** Correlations between HLA-DRA and TIICs estimated by TIMER and EPIC algorithms in pan-cancer. The color indicates the correlation coefficient. The asterisks indicate significant differences assessed by Pearson analysis. **D** Schematic protocol of validation using the pan-cancer TMA cohort. **E** Representative images revealing HLA-DRA and PD-L1 expression in various tumor types. Magnification, 200×. **F** Correlation between HLA-DRA and PD-L1 expression in the pan-cancer TMA cohort

Our data illustrated that HLA-DRA was associated with an inflamed TME and could identify immuno-hot tumors in NSCLC. However, the immunological role of HLA-DRA in other cancer types was unclear. Next, we analyzed the correlations between HLA-DRA and chemokine, MHC, receptor, immunoinhibitors, and immunostimulators. Except for several cancer types, HLA-DRA was positively correlated with the expression levels of these immunomodulators (Fig. 6A). Moreover, HLA-DRA was negatively correlated with tumor purity but positively correlated with TIIC levels in most cancer types (Figs. 6B-C). HLA-DRA was also positively correlated with the expression levels of immune checkpoints in pan-cancer (**Supplementary Figs. S****2**). We next collected a pan-cancer TMA cohort to validate the above findings (Fig. 6D). The results showed that HLA-DRA was highly correlated with PD-L1 expression in the current cohort (Fig. 6E-F). Taken together, the data suggested that HLA-DRA is a pan-cancer classifier for immuno-hot tumors except for a few tumor types.

## Discussion

Anti-PD-1 immunotherapy exhibited encouraging efficacy for advanced NSCLC, but not all patients could benefit from immunotherapy. Predictive signatures to identify the dominant population are urgent for the field. TME, consisting of multiple cell types and the interplay between these cells via cytokines, chemokines, and growth factors [33], lays the basis for determining whether immunotherapy is effective [34]. According to the features of TME, tumors can be classified into immuno-cold or immuno-hot. Immuno-cold tumors are featured with immunosuppressive TME and insensitive immunotherapy, but immuno-hot tumors represent the high response to immunotherapy, which is accompanied by active T cell infiltration [35]. Therefore, distinguishing immuno-hot or immuno-cold tumors using practical biomarkers is a significant strategy to demarcate the response to immunotherapy.

In the current research, we obtained three public RNA-seq datasets from immunotherapy cohorts to identify novel biomarkers for immunotherapy, and reported that CD2, HAVCR2, HLA-C, HLA-DRA, HLA-E, KLRK1, and TYROBP might be novel biomarkers for immunotherapy in NSCLC. In view of expression, atlas of cell subtypes, and prognostic values, we selected HLA-DRA for further investigation and validation. We found that HLA-DRA was associated with an inflamed TME and identified immuno-hot tumors in NSCLC. Additionally, HLA-DRA could predict the response to immunotherapy from the point of multiple signatures and in our recruited cohort. Moreover, pan-cancer investigation and validation revealed that HLA-DRA was a pan-cancer classifier for immuno-hot tumors.

Previous research has proved that HLA-DR participates in the inhibition of tumor growth. HLA-DR presents tumor-associated antigens (TAA) that are recognized by CD4+ T cells, which then produce cytokines, including interleukins and interferon-γ, to suppress tumor growth [36, 37]. HLA-DRA is a subunit of HLA-DR, which plays a critical role in human cancers. Overexpression of HLA-DRA was reported in hepatocellular cancer [38], colorectal cancer [39], and cervical cancer [40], while it was decreased in breast cancer [41]. In this report, we found that HLA-DRA was significantly decreased in NSCLC. In addition, HLA-DRA has also been shown to function as a prognostic biomarker for clinical outcomes [42, 43]. Nevertheless, no research has uncovered the predictive role of HLA-DRA for immunotherapy in NSCLC.

In addition to the observational results from the public and in-house cohorts, we found that HLA-DRA was associated with multiple immunotherapy-related pathways. HLA-DRA was positively correlated with IFN-γ signature, APM signal, and hypoxia, while was negatively correlated with FGFR3-coexpressed genes, PPAR-γ network, WNT/β-catenin network, and VEGFA pathway. IFN-γ, APM, and hypoxia pathways were correlated with high response to immunotherapy [44, 45], while FGFR3, β-catenin, PPAR-γ, and VEGFA pathways were activated in the non-inflamed tumors [46, 47]. The interplays between HLA-DRA and IFN-γ signature as well as the APM signal were clear [48, 49]. For example, IFN-γ could increase the expression of the HLA-DRA [49]. However, whether HLA-DRA could regulate other immunotherapy-related pathways needs to be further studied.

## Conclusions

This study identified potential biomarkers to predict the response to immunotherapy. After systematic analysis, HLA-DRA could a promising biomarker that is associated with an inflamed TME and may predict the response to immunotherapy in NSCLC. Furthermore, the pan-cancer analysis revealed that HLA-DRA identifies high immunogenicity. Overall, HLA-DRA might be a promising biomarker for guiding immunotherapy in NSCLC.

## Supplementary Information

**Additional file 1.**

## Data Availability

The original contributions presented in the study are included in the article/Supplementary Material. Further inquiries can be directed to the corresponding authors. In addition, original data for bioinformatics analysis could be downloaded from corresponding platforms.

## Abbreviations

NSCLC: Non-small cell lung cancer
TKI: Tyrosine kinase inhibitor
PFS: Progression-free survival
OS: Overall survival
ICB: Immune checkpoint blockade
GEO: Gene Expression Omnibus
HLA-DRA: Human leukocyte antigen-DR alpha
TCGA: The Cancer Genome Atlas
RNA-seq: RNA-sequencing
DEG: Differentially expressed gene
BP: Biological process
scRNA-seq: Single-cell RNA-sequencing
TME: Tumor microenvironment
TIIC: Tumor-infiltrating immune cell
IPS: Immunophenoscore
TCIA: The Cancer Immunome Atlas
TMA: TISSUE microarray
PR: Partial remission
SD: Stable disease
IHC: Immunohistochemistry
IRS: Immunoreactivity score
LUSC: Lung squamous cell carcinoma
LUAD: Lung adenocarcinoma
TAA: Tumor-associated antigens
