# Correction to: HLA Class II molecule HLA-DRA identifies immuno-hot tumors and predicts the therapeutic response to anti-PD-1 immunotherapy in NSCLC

**DOI:** 10.1186/s12885-026-16779-5

**Published:** 2026-09-17

**Authors:** Jie Mei, Guanyu Jiang, Yundi Chen, Yongrui Xu, Yuan Wan, Ruo Chen, Feng Liu, Wenjun Mao, Mingfeng Zheng, Junying Xu

**Affiliations:** 1 Department of Oncology, The Affiliated Wuxi People’s Hospital of Nanjing Medical University, Wuxi, 214023 China; 2 Department of Cardiothoracic Surgery, The Affiliated Wuxi People’s Hospital of Nanjing Medical University, Wuxi, 214023 China; 3 The Pq Laboratory of BiomeDx/Rx, Department of Biomedical Engineering, Binghamton University, Binghamton, NY 13902 USA

**Correction to: BMC Cancer 22**,** 738 (2022)**

**https://doi.org/10.1186/s12885-022-09840-6**

Following publication of the original article [1], the authors identified an error in Fig. 5E, wherein the PR HLA-DRA and PR PD-L1 panels were inadvertently duplicated due to erroneous layer arrangement in Adobe Illustrator. The revised Fig. 5E, now displaying the correct image for the PR PD-L1 panel, is provided below. We confirm that the correction does not affect the study’s conclusions.

**Incorrect Fig. 5**

**Fig. 5:**
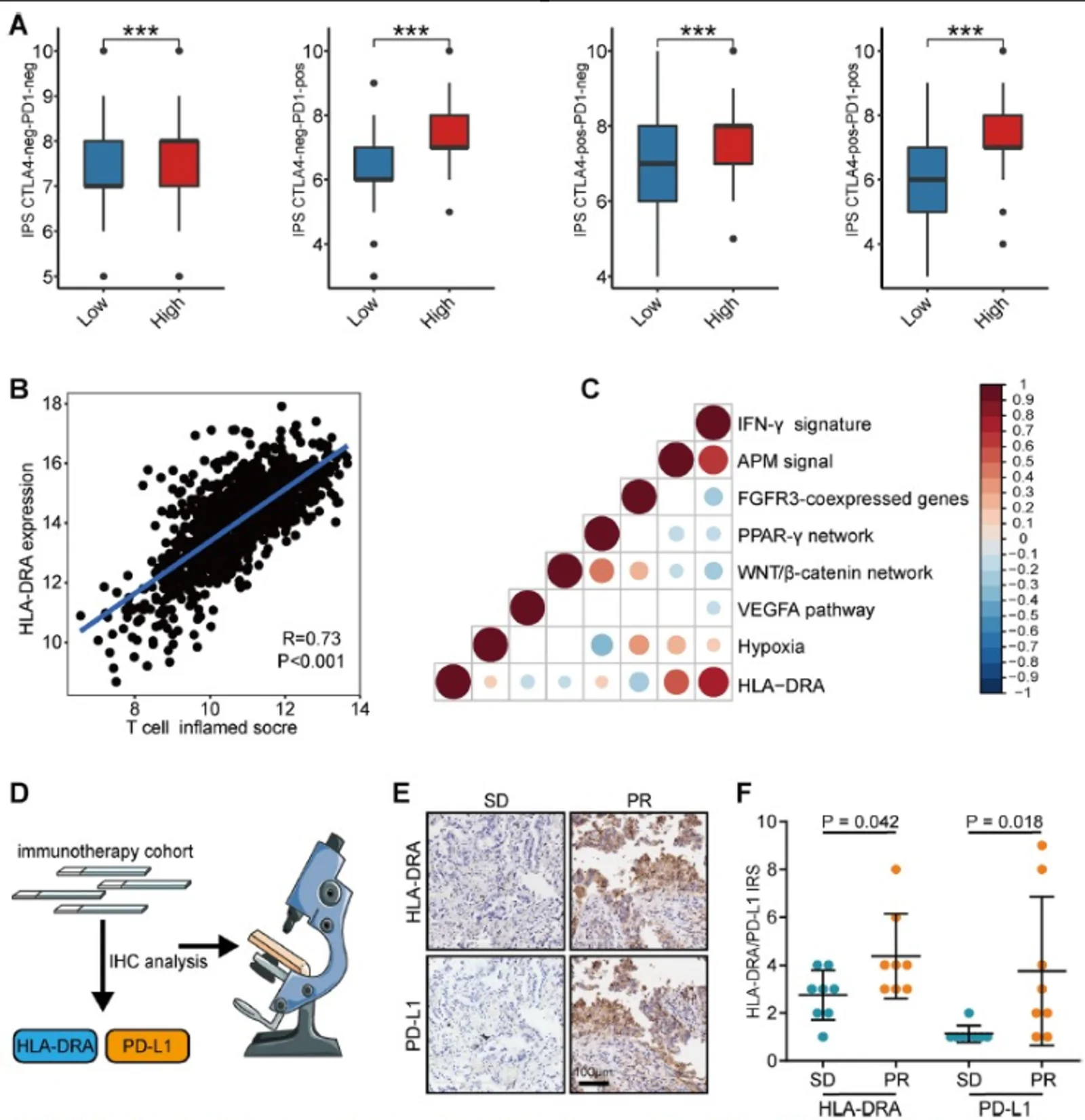
HLA-DRA predicts immunotherapeutic response of immunotherapy. **A** Differences in levels of IPS in the high and low HLA-DRA groups in NSCLC. **B** Correlation between HLA-DRA and T cell inflamed score in NSCLC. **C** Correlation between HLA-DRA and immune-related pathways in NSCLC. **D** Schematic protocol of validation using the immunotherapy cohort. **E** Representative images revealing HLA-DRA and PD-L1 expression in NSCLC tissues with different immunotherapeutic responses. Magnification, 200×. **F** Expression levels of HLA-DRA and PD-L1 in NSCLC patients with immunotherapeutic responses

**Correct Fig. 5**

**Fig. 5:**
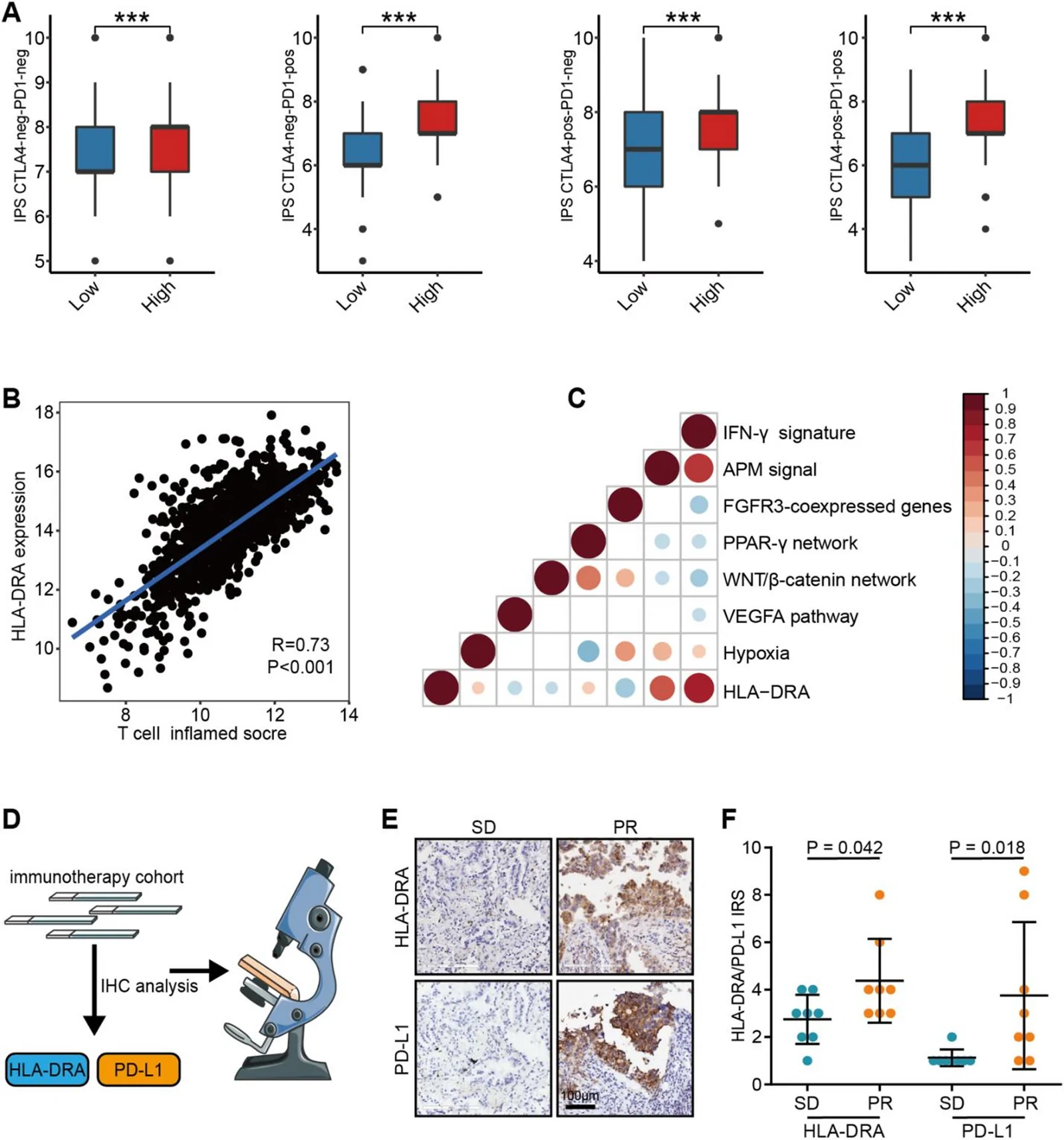
HLA-DRA predicts immunotherapeutic response of immunotherapy. **A** Differences in levels of IPS in the high and low HLA-DRA groups in NSCLC. **B** Correlation between HLA-DRA and T cell inflamed score in NSCLC. **C** Correlation between HLA-DRA and immune-related pathways in NSCLC. **D** Schematic protocol of validation using the immunotherapy cohort. **E** Representative images revealing HLA-DRA and PD-L1 expression in NSCLC tissues with different immunotherapeutic responses. Magnification, 200×. **F** Expression levels of HLA-DRA and PD-L1 in NSCLC patients with immunotherapeutic responses
