# Supplementary material for: HLA class II molecule HLA-DRA identifies immuno-hot tumors and predicts the therapeutic response to anti-PD-1 immunotherapy in NSCLC
Source: BMC Cancer. 2022 Jul 6;22:738. doi: 10.1186/s12885-022-09840-6 (PMC9258174; doi:10.1186/s12885-022-09840-6)
Supplement: Supplementary file 1 — Additional file 1. [file 12885_2022_9840_MOESM1_ESM.docx]

**Supplementary Figures**

**
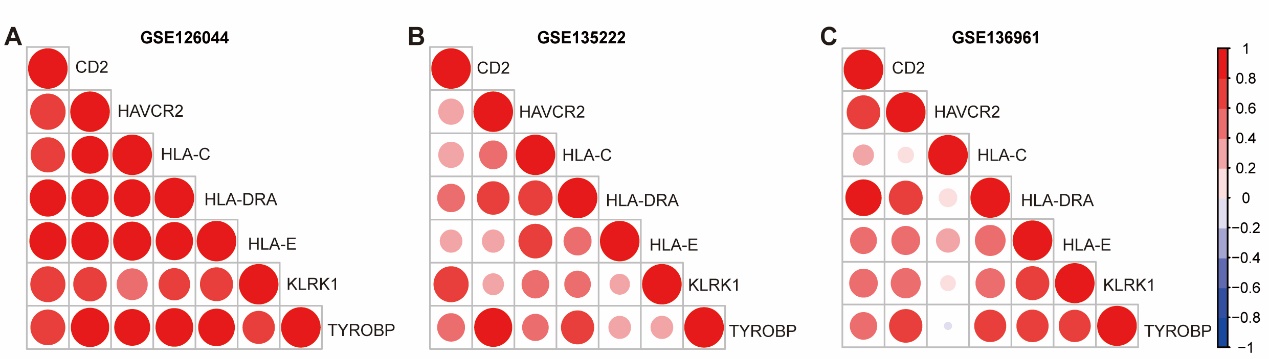
**

**Figure S1. Correlations between seven potential biomarkers.**

(A) Correlations in the GSE126044 dataset. (B) Correlations in the GSE13522 dataset. (C) Correlations in the GSE136961 dataset.

**
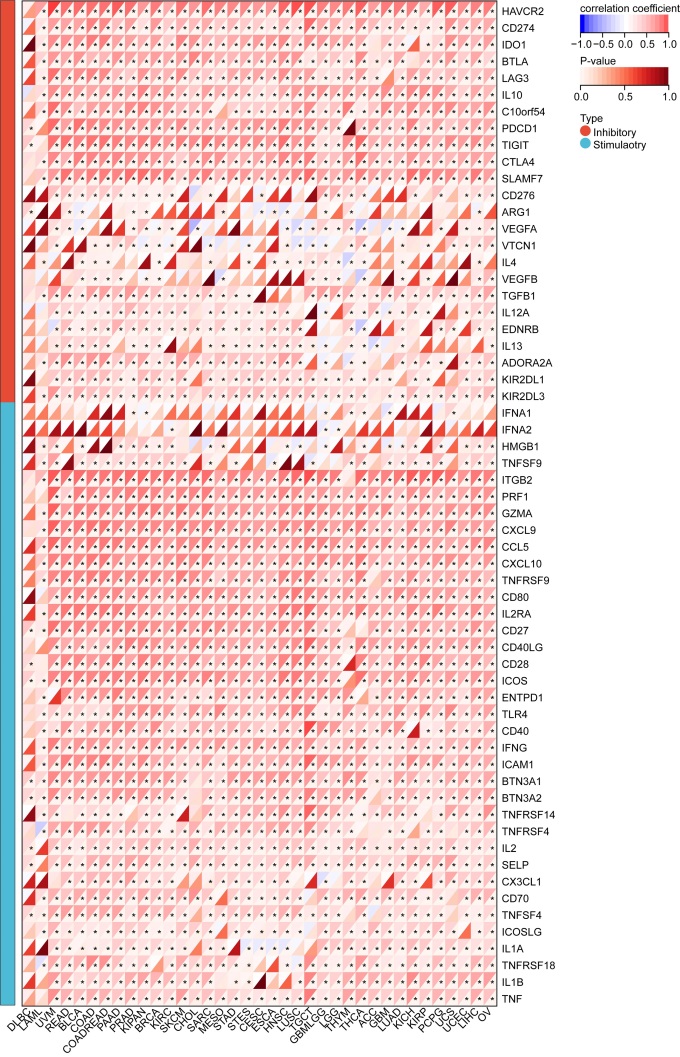
**

**Figure S2. Correlations between HLA-DRA and immune checkpoints expression in pan-cancer.** The color indicates the correlation coefficient. The asterisks indicate significant differences assessed by Pearson analysis.

**Supplementary Tables**

**Table S1. Summary of abbreviations in the TCGA dataset.**

| Abbreviation | Full name |
| --- | --- |
| ACC | adrenocortical carcinoma |
| BLCA | bladder urothelial carcinoma |
| BRCA | breast invasive carcinoma |
| CESC | cervical & endocervical cancer |
| CHOL | cholangio carcinoma |
| COAD | colon adenocarcinoma |
| DLBC | lymphoid neoplasm diffuse large B-cell lymphoma |
| ESCA | esophageal carcinoma |
| GBM | glioblastoma multiforme |
| HNSC | head and neck squamous cell carcinoma |
| KICH | kidney chromophobe carcinoma |
| KIRC | kidney renal clear cell carcinoma |
| KIRP | kidney renal papillary cell carcinoma |
| LAML | acute myeloid leukemia |
| LGG | brain lower grade glioma |
| LIHC | liver hepatocellular carcinoma |
| LUAD | lung adenocarcinoma |
| LUSC | lung squamous cell carcinoma |
| MESO | mesothelioma |
| OV | ovarian serous cystadenocarcinoma |
| PAAD | pancreatic adenocarcinoma |
| PCPG | pheochromocytoma and paraganglioma |
| PRAD | prostate adenocarcinoma |
| READ | rectum adenocarcinoma |
| SARC | sarcoma |
| SKCM | skin cutaneous melanoma |
| STAD | stomach adenocarcinoma |
| TGCT | testicular germ cell tumors |
| THCA | thyroid carcinoma |
| THYM | thymoma |
| UCEC | uterine corpus endometrial carcinoma |
| UCS | uterine carcinosarcoma |
| UVM | uveal melanoma |

**Table S2. Detailed information on immunotherapy-related gene signatures.**

| Pathway | Reference | Genes |
| --- | --- | --- |
| IFN-γ signature | PMID: 28650338 | TIGIT, CD27, CD8A, PDCD1LG2, LAG3, CD274, CXCR6, CMKLR1, NKG7, CCL5, PSMB10, IDO1, CXCL9, HLA-DQA1, CD276, STAT1, HLA-DRB1, HLA-E |
| APM signal | PMID: 31563503 | B2M, HLA-A, HLA-B, HLA-C, TAP1, TAP2 |
| FGFR3-coexpressed genes | PMID: 31563503 | FGFR3, TP63, IRS1, SEMA4B, PTPN13, TMPRSS4 |
| PPARG network | PMID: 27197067 | PPARG, IGFBP3, GDF15, MYH14, IHH, OCLN, AQP3, SCNN1G, PLIN5, KRT19, GPT, CYP4B1, UGT1A7, DGAT2, KRT20, SNCG, GSTA1, ACADL, BDH1, HMGCS2, LIPE |
| WNT/β-catenin network | PMID: 27197067 | CTNNB1, TFF1, HAPLN1, IHH, WNT7B, BMP7, SEMA5A, SCN5A, ERBB3, TSPAN8, EPCAM, TH, GPX2, GAD1, HSD17B2, KRT7, NOX1, CYB5A, CYP4F12, ID4, SIM2, MECOM, MSX2, KLF5, SMAD6, POU5F1, FOXQ1, GATA2, GATA3, EMX2 |
| VEGFA pathway | PMID: 24793239 | VEGFA |
| Hypoxia | PMID: 31563503 | CAV1, COL5A1, ITGA5, P4HA2, SLC16A1, TGFBI, DPYSL2, SRPX, TRAM2, SYDE1, LRP1, PDLIM2, SAV1, AHNAK2, CAD, CYP1B1, DAAM1, DSC2, SLC2A3, FUT11, GLG1, GULP1, LDLR, THBS4 |

**Table S3. Detailed information on BP enrichments of 7 candidates.**

| ID | Term | Count | P value | Genes |
| --- | --- | --- | --- | --- |
| GO:0045087 | innate immune response | 5 | <0.001 | KLRK1, TYROBP, HLA-C, HAVCR2, HLA-E |
| GO:0050776 | regulation of immune response | 4 | <0.001 | KLRK1, TYROBP, HLA-C, HLA-E |
| GO:0019882 | antigen processing and presentation | 3 | <0.001 | HLA-DRA, HLA-C, HLA-E |
| GO:0060333 | interferon-gamma-mediated signaling pathway | 3 | <0.001 | HLA-DRA, HLA-C, HLA-E |
| GO:0050830 | defense response to Gram-positive bacterium | 3 | <0.001 | KLRK1, HAVCR2, HLA-E |
| GO:0002250 | adaptive immune response | 3 | 0.001 | KLRK1, HAVCR2, HLA-E |
| GO:0002281 | macrophage activation involved in immune response | 2 | 0.003 | TYROBP, HAVCR2 |
| GO:0002480 | antigen processing and presentation of exogenous peptide antigen via MHC class I, TAP-independent | 2 | 0.003 | HLA-C, HLA-E |
| GO:0030101 | natural killer cell activation | 2 | 0.007 | CD2, KLRK1 |
| GO:0032753 | positive regulation of interleukin-4 production | 2 | 0.007 | HAVCR2, HLA-E |
